# Lysol

**Published:** 1893-08

**Authors:** Henri De Parville, Carlton A. Kingsbury

**Affiliations:** Westfield, N. Y.


### LYSOL.

By Dr. HENRI de PARVILLE.

(Translated from *Les Annales*)

By Mrs. CARLTON A. KINGSBURY, of Westfield, N. Y.

For some time, one has heard on all sides of lysol. What is it? Lysol promises to become the king of antiseptics. What is an antiseptic? A substance which kills microbes! What are microbes? Microscopic organisms which, in favorable conditions, multiply with frightful rapidity; only one germ is necessary to multiply, in a few hours, into thousands and millions. From the labors of Pasteur, of Davaine, and of their disciples, it has been demonstrated that epidemic diseases and contagious affections have their origin in a microbe; each malady has its specific microbe, which takes the name, according to kind—bacteria, vibrio, micrococcus, etc. In case of crime, one says: "Search for the woman!" In case of disease, one says today: "Search for the microbe!" And in fact, it is found in cases where it is least suspected. Carbuncles, for example, have their origin in a microbe, and carbuncles are contagious; when a person has one, others are sure to follow. Tetanus has its cause in a microbe, etc. But the great epidemic diseases are, above all, microbic affections; for example, cholera, yellow fever, typhoid and septic fevers, measles, small-pox, diphtheria, contagious pneumonia, grippe, tuberculosis, and its cousin germain, scrofula. Even in animal diseases—search for the microbe!

These new doctrines, founded upon certain discoveries, upon severely contested facts, have wrought a complete revolution in medical theories and modify considerably, therapeutics. One may declare that the whole progress of surgery has been brought about by the antiseptic; a great number of victories won by medicine are due to an antiseptic. It rules, rightly, in all large cities, in the medical centers of Europe, in England, Germany, France, Russia—everywhere. The antiseptic has saved from death a large number of sick people. Since antiseptic dressings for wounds have been used, it is estimated that the mortality in surgical operations has been lowered from 50 per 100 to 5 per 100. In accouchement, it has fallen from 20 per 100 to 3 per 100. Why? Because antiseptics kill the deadly microbes! Into a badly protected wound, atmospheric germs penetrate. These microörganisms fall upon the bare flesh, find an excellent place for development, penetrating the system and infecting it. One suffices to people the tissues and bring death. The antiseptic placed upon the wound is a vigilant guard. It kills the microbe which presents itself; it prevents it from penetrating the flesh and pursuing its work of destruction.

We have not, until now, believed in the internal antiseptic; that is, in antiseptics introduced into the body. The problem becomes complicated, and the recent personal observation of Doctor A. Robin, who saw contagious pneumonia declare itself in a house saturated with corrosive sublimate, but serves to give us more confidence in the internal antiseptic. The microbe enters our houses, finds a lodging place, and grows and multiplies. The more reason for guarding, with particular care, the door of entrance, and using in large quantities the external antiseptic. This, at least, has been proved, and its efficacy is not to be doubted. We have spoken, recently, of the utility in combating grippe, colds, epidemic maladies, by the use of antiseptic douches for the nose and mouth, the ordinary receptacles of the most deadly microbes. In truth, we respire at the rate of one-half liter of air at a respiration; this gives, moderately, 360 liters per hour, and 9,000 liters, nearly ten cubic meters, in twenty-four hours. Each liter draws in millions of microbes. All these microbes are not noxious, but they are often injurious on account of their number; hence, the necessity of killing them before an abrasion, an inflammation, opens to them the door of the organism. Microbes can enter the body through perspiration not wiped off. One can readily see that the need of cleanliness, and of general hygiene, is of vast importance. Briefly, it is necessary to repeat that we are constantly the objects of the attacks of these microörganisms; simple common sense recommends us to multiply our efforts for defense. This defense is the rational and abundant use of antiseptics.

Antiseptic! the word is an easy one to speak. But antiseptics are abundant; at least, one reads so. We are not rich in antiseptics suitable to handle, not dangerous, yet efficacious. Then, too, certain antiseptics which produce good results in one case, do not in another. An antiseptic which can be generally used is not easy to find. Many have been in use for years, or are dangerous, or have lost their efficacy. It is superfluous to pass them in review. Phenic acid, to mention the best known, is not safe to place in the hands of everyone; it is not neutral, it is corrosive; it has burned more than one person; it has even poisoned some; it is, moreover, an antiseptic of only the fifth or sixth class. The essence of Ceylon cinnamon is, differently, as powerful as phenic acid. It kills the most resisting microbes in eight minutes after contact, while it takes, at least, twenty-four hours to kill them with phenic acid. In order to judge of the action of an antiseptic, it is indispensable that one does not content one's self with "hear-say" instead of experimenting with it; of cultivating the most dangerous microbes and seeing how they act in the presence of the antiseptic used. The best is, evidently, that which kills the most rapidly; when, in operating thus, it has been found that the most efficacious substance was, unquestionably, corrosive sublimate. Unfortunately, corrosive sublimate is a violent poison. Although one may use it in very weak solutions, there is certainly danger in introducing it into common practice. People who have made mistakes in swallowing a solution of sublimate have died very quickly. Thymol is costly; boric acid is weak. If one wishes to gain an idea of the destructive power of sublimate, of thymol, of boric acid, we will state that with three centigrammes of sublimate in solution, microbic development is prevented; with thymol, it takes fifty centigrammes; with boric acid, 750 centigrammes.

After sublimate, which is rejected for general use, the powerful antiseptics meet in the derivations of coal-tar, in the phenols, the various creasotes, creasol (cresylic acid), and its varieties, salicylic acid, eucalyptol, naphthol, etc. By order of power, one has, first, creasol (100 centigrammes arresting development of bacteria); then salicylic acid (150 centigrammes arresting development). Finally, eucalyptol (160 centigrammes), and phenol (500 centigrammes). Commercially, on account of price, phenol and creasol are alone obtainable. And one prefers the strongest, *i. e.*, creasol.

This creasol of commerce, produced actively from creasote of coal, deprived of its phenic acid possesses, definitely, a strong antiseptic, since it suffices in a solution of 0.30 per cent. of water to obtain an action equal to a disinfecting liquid of sublimate at 1 per 4,000. Unfortunately, creasol is insoluble in water. Now, it is very necessary that an antiseptic should be soluble, because water is the medium of disinfecting action; it is everywhere; it brings the "deadly microbes" into contact with the tissues. In order to utilize creasol, one must find the means of rendering it soluble. Much research has been made in this direction. Laplace, Frankel, Heyden, and others, have saponified bodies of the aromatic series by diverse alkaline bases; but these products oxidate in the air, lose their antiseptic power, stain the hands and instruments used. There has been found a new and economical mode of rendering creasol soluble in water. In France, this brief process has been achieved by a society which sell this product at a price accessible to all. This product is the famous lysol.

Chemically, they prepare the lysol of commerce by utilizing creasol obtained by rectifying, between 195 and 205°, the heavy oils of coal tar. And this obtains a crude creasol, containing a little creasol, xylenol, gaïacol, etc. Then this crude creasol is rectified to render it pure. It is mixed in the proportion of fifty parts to fifty other parts of an alkali, and with fat parts. By cooking this, a liquid is obtained, of a brown color, absolutely *soluble* in water. The characteristic of this product is double: First, its complete solubility in water; then its constant chemical composition. This is a fixed product whose properties are invariable today and tomorrow.

There remains now but the question of its power. Theoretically, it is necessary to employ twice as much of the lysol as of creasol to obtain the same disinfection. But it is its solubility. Always, as has been demonstrated, the lysol is found to be as powerful as the creasol. In Germany, in fact, its usage has become common since the researches made by M. Schottelius, Professor at Friburg; by M. de Gerlach, Chief of the Hygienic Department in the Laboratory of the Institute at Wiesbaden; by

Mm. Whemer, Cramer, Hanel, Hang, and others. Now, the results are remarkable. M. de Gerlach, in operating upon the spores of the bacteria of a carbuncle, bacteria extremely resistant, which had not been affected by an action of fifty days of phenic acid at 5 per 100, has observed the following facts with a solution scarcely less strong of lysol: In an hour, development retarded; in twelve hours, colonies stopping growth; in seventy-five hours, death. This culture inoculated into a rabbit left it unharmed. One can sum up the experiments of Mm. Gerlach and Schottelius, in saying that with a dose of three grammes par liter of lysol they have assured the disinfection of the most resistant septic matters. One is authorized in considering, on account of its strength, lysol as creasol rendered soluble. It is, then, right to regard the product as a general antiseptic, actually the most powerful, the most economical, and the most easy of access to every one.

In fact, it is established as a medicine, invariable in composition, as we have said; it is not an irritant, contains no acid; does not attack the tissues, as in time does sublimate, phenic acid, and others. It is not poisonous. Then, let it become of common use. It always mixes easily with water. It is used in the ordinary dose of three grammes par liter, and it can be used to five per cent. Everything depends on the use to which it is put. The uses of antiseptics are, in fact, very numerous. The microbe-killing power of lysol designs it, naturally, for dressing of wounds, for cicatrizations, slight abrasions, etc. In the point of view of general hygiene, it is useful to have at hand for disinfecting pest-houses, apartments, cabinets, spittoons, animal cars, holds of ships, steamers, and everywhere where disinfectants are required. To cleanse wounds, a solution is used, composed of between 0.30 to one per cent.; for gynecologia and like diseases, a solution of one to three per cent. is used. For disinfecting the thousand contaminations—stables, cess-pools, moldy substances growing on damp walls, a solution of three to five per cent. is used. In these conditions, a liter of lysol, of a commercial price sufficiently small, makes, according to the use required, from twenty to 300 liters of disinfecting solution.

For the toilet one does not generally go beyond 0.50 to one. This dose of lysol (thanks to the soapy properties of creasol) has no irritating action; it serves for a dentifrice, for an eye-wash, etc. Its characteristic odor is somewhat like creasote and tar, but this is modified for those to whom the tar smell is offensive, by adding perfumes, as mint, cinnamon, etc. It is most useful in the service of newly-born infants, and for children. Finally, it is excellent as an aid to agriculture. It destroys insects, parasitic vegetation of plants, aphides, ants, oïdium, doryphora, etc.

We have searched for a strong manageable antiseptic, free from danger, neither corrosive nor poisonous. We have obtained it now, because it seems that lysol answers entirely the requirements ; then, let us not wait until an epidemic attacks us ; and let us not hesitate to introduce into our homes, for constant use, this antiseptic, that is to say, hygienic preservation.

If we have insisted a little on this subject, it is because we believe more and more in the necessity of the antiseptic. It will gain for us many years, many human lives ; and what great need we have of saving ourselves from disease ! Let us defend ourselves and our homes against the homicidal microbe !

